# High‐Entropy Ceramic‐Enhanced Deformable Triboelectric Nanogenerator for Noncontact Biomechanical and Raindrop Energy Harvesting

**DOI:** 10.1002/adma.74157

**Published:** 2026-07-25

**Authors:** Shengyou Li, Wei‐Chen Peng, Syun‐Hong Chou, Zhi‐Xian Yan, Jiann‐Yeu Chen, Dun‐Jie Jhan, Wei‐Chun Yang, Shi‐Hong Chen, Yi‐Lin Huang, Cheng‐Hung Tsai, Ming‐Yen Lu, Heng‐Jui Liu, Cheolmin Park, Ying‐Chih Lai

**Affiliations:** ^1^ Department of Materials Science and Engineering National Chung Hsing University Taichung Taiwan; ^2^ Department of Materials Science and Engineering Yonsei University Seoul Republic of Korea; ^3^ Department of Materials Science and Engineering National Yang Ming Chiao Tung University Hsinchu Taiwan; ^4^ Innovation and Development Center of Sustainable Agriculture I‐Center for Advanced Science and Technology National Chung Hsing University Taichung Taiwan; ^5^ Department of Materials Science and Engineering National Tsing Hua University Hsinchu Taiwan; ^6^ Department of Physics National Chung Hsing University Taichung City Taiwan

**Keywords:** High‐entropy ceramics, noncontact triboelectric nanogenerator, raindrops energy harvesting, wearable power source

## Abstract

To address interfacial friction losses associated with physical contact separation in triboelectric nanogenerators (TENGs), noncontact or liquid‐solid‐contact TENGs have recently been developed. However, the limited performance of noncontact TENGs has hindered their further advancement. This study proposed a large‐area deformable high‐entropy ceramic (HEC)‐enhanced noncontact TENG (HEC‐TENG) by integrating an HEC‐doped silicone triboelectric layer with a charge‐storage graphitic textile and a stretchable carbon black electrode. This strategy establishes a comprehensive tribo‐charge regulation mechanism, including charge generation, capture, transport, and retention, resulting in a noncontact output of 90 V and 450 µA m^−2^ at a separation distance of 1 mm, while maintaining a stretchability exceeding 230%. Notably, by leveraging the synergistic effects of HEC doping and the trilayer composite configuration, the HEC‐TENG achieved a maximum voltage of 466 V for droplet‐based energy harvesting. Furthermore, wearable power sources and large‐area rainwater‐harvesting systems enabled by HEC‐TENGs were demonstrated. Overall, this work validates the universal enhancement effect of HECs when employed as triboelectric layers in both noncontact and liquid‐solid contact TENGs, establishing a representative paradigm for the development of wearable HEC‐based TENGs. These findings provide new insights into future wearable self‐powered electronics and distributed energy‐harvesting systems.

## Graphical abstract

A large‐area, deformable high‐entropy‐ceramic (HEC)‐doped triboelectric nanogenerator with a trilayer architecture is developed, consisting of an HEC‐doped triboelectric layer, a graphite‐like textile charge‐storage layer, and stretchable carbon black electrodes. HEC incorporation significantly enhances triboelectric output in a fully stretchable design, enabling noncontact biomechanical and droplet energy harvesting for wearable and large‐area distributed power source applications.

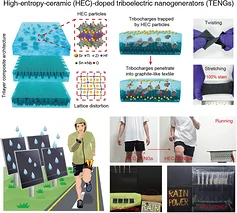

## 1 Introduction

Wearable energy harvesting technologies have emerged as indispensable components for the future development of flexible/stretchable electronics and distributed energy power sources for the Internet of Things (IoTs). They can efficiently capture diverse forms of ambient energy, such as mechanical [1, 2, 3], raindrop [4, 5, 6], solar [7], moist [8, 9], thermal [10], and magnetic energies [11], and convert these forms into electrical power to drive wearable electronics or IoT devices, thereby eliminating the reliance on conventional bulky batteries [12, 13, 14]. Triboelectric nanogenerators (TENGs), which operate based on the principles of triboelectrification and electrostatic induction, have attracted considerable attention because of their ability to effectively harvest diverse forms of ambient mechanical energy and convert them into electricity [1, 12, 15, 16, 17]. Owing to their unique advantages, such as material diversity, mechanical flexibility and stretchability, adaptability, high output performance, biocompatibility, low cost, and scalability, TENGs have been extensively explored for applications in wearable/implantable power sources [2, 3, 18], electronic skins [19, 20], and large‐area distributed energy harvesting systems in recent years [21, 22].

However, conventional TENGs typically rely on the contact‐separation process between two triboelectric materials, which leads to interfacial contamination, surface wear, and inefficient energy conversion [3, 23]. To address these challenges, the development of noncontact TENGs has been proposed to alleviate interfacial friction‐induced losses and thereby enable long‐term stable operation [24]. Nevertheless, the performance of noncontact TENGs is often inferior to that of contact‐separation TENGs, primarily because of their limited charge‐trapping capability at the triboelectric interface and poor triboelectric charge retention and reliability during noncontact operation. On this basis, researchers have reported enhancing the charge‐trapping capability of noncontact TENGs by incorporating high‐permittivity materials (e.g., CaCu_3_Ti_4_O_12_, BaTiO_3_ or Bi_2_Se_3_) into silicone‐based triboelectric layers [23, 25, 26]. For instance, CaCu_3_Ti_4_O_12_‐based TENG can generate 15.1 V and 420 nA at 0.5 mm separation distance [26]. In addition, the design of a triple‐layer device configuration (triboelectric layer‐conductive layer‐electrode), in which the intermediate layer functions as a charge storage layer, has been proposed to improve the charge‐retention capacity [3, 23]. For example, Rana et al. introduced conductive MXene nanosheets into a TENG architecture to store tribocharges, thereby extending the charge retention time to 4320 min [27]. By contrast, droplet‐based energy generators (DEGs) or liquid‐solid contact TENGs, which operate via the formation of interfacial electric double layers (EDLs) induced by the spreading and contraction of droplets on hydrophobic triboelectric surfaces, have recently gained significant attention [5, 6, 28]. Owing to the soft, damage‐free contact between droplets and solids, as well as the self‐cleaning properties of water flow, DEGs can also effectively avoid frictional losses and interfacial contamination compared with conventional TENGs. However, current studies on DEGs have primarily focused on enhancing the performance, whereas the stretchable design of devices has been largely overlooked, rendering most reported DEG architectures unsuitable for wearable applications [4, 6].

To address the bottlenecks of current devices, high‐entropy ceramics (HECs), as emerging high‐dielectric materials, have attracted increasing interest owing to their unique physical properties and multicomponent solid‐solution structures [29, 30]. The coexistence of multiple constituent elements within a single lattice induces pronounced local lattice distortion, effectively enhancing polarization behavior. Moreover, synergistic interactions among multiple cations, particularly transition metal species, generate abundant deep trap states and point defects such as oxygen vacancies, thereby enriching the band structure and strengthening surface electrostatic regulation [31, 32]. These advantages demonstrate the potential of HECs as high‐performance dielectric materials for next‐generation TENG or DEGs. For instance, Wang et al. developed an HEC‐doped DEG, in which (MgCoNiZn)_0.88_Cu_0.12_O‐doped polydimethylsiloxane (PDMS) served as an intermediate layer to suppress triboelectric charge decay, resulting in a high open‐circuit voltage (V_oc_) of 525 V [33]. In addition, Zhou et al. reported a fluorinated ethylene propylene (FEP)/(FeCoNiCrMn)_3_O_4_‐doped PDMS/Cu DEG that delivered a V_oc_ of 420 V and a short‐circuit current (I_sc_) of 0.23 mA, achieving nearly threefold higher output performance compared with the FEP/Cu DEG counterpart [34]. However, noncontact‐mode TENGs generally require an enhanced dielectric constant and strengthened interfacial polarization within the triboelectric layer to promote triboelectric charge trapping capability. In the previously reported HEC‐doped DEG devices, the HEC materials were used only as intermediate charge‐storage layers and did not contribute to enhancing triboelectric charge generation. In addition, although fluorinated polymer films such as fluorinated ethylene propylene can provide high interfacial charge transfer capability as triboelectric layers, their intrinsic nonstretchable nature makes it difficult to realize soft, deformable, and stretchable TENG devices, thereby limiting their applications in wearable energy harvesting.

Here, we present a large‐area, stretchable, noncontact TENG based on HECs and graphite‐like carbonized textiles with excellent hybrid‐source energy‐harvesting performance and outstanding mechanical deformability. Hereafter, the device is referred to as the HEC‐TENG. It serves as a wearable noncontact energy harvester and can be extended into a distributed IoT energy‐harvesting system. The HEC‐TENG combines high‐output energy harvesting from biomechanical motions and droplet impacts with excellent stretchability of up to 230% strain and prolonged tribocharge retention of 470 min, enabling its integration into garments for noncontact human‐motion energy harvesting. A comprehensive comparison between this study and previously reported noncontact TENGs and droplet energy harvesting devices is summarized in Table S1. The HEC‐TENG comprises a HEC‐doped silicone rubber (SR) triboelectric layer coated on a graphite‐like textile (GT) charge storage layer and a carbon black (CB)/SR stretchable electrode layer. Its outputs exhibit a V_oc_ of 90 V and a short‐circuit current density (J_sc_) of 450 µA m^−^^2^ under noncontact operation with 1 mm distance. When harvesting droplet energy, the output V_oc_ and maximum power reach 466 V and 150 µW, respectively. Notably, its output is approximately four times higher than the pure SR‐DEG device. The superior performance of HEC‐TENG can be attributed to three key factors. First, the roughened surface of HEC‐doped SR layer effectively enhances the tribo‐charge generation. Second, the uniformly dispersed HEC particles within the SR matrix significantly enhance dielectric polarization and charge trapping. Third, the GT layer further promotes long‐term triboelectric charge retention. Collectively, these synergistic effects establish a comprehensive tribocharge regulation process, including charge generation, transport, retention, and storage, thereby enhancing outputs. In harvesting both noncontact mechanical motions and droplet impacts, the generated electrical energy from the HEC‐TENG is directly utilized to power practical electronics, such as watches and calculators. In summary, this study demonstrates the remarkable potential of HEC doping in enhancing the output of noncontact TENGs, while simultaneously addressing the stretchability challenge of HEC‐based TENGs. Furthermore, the tri‐layer composite architecture exhibits an additional enhancement effect in the operation of droplet energy harvesting. These findings provide new insights for future wearable self‐powered electronics and distributed energy harvesting systems.

## 2 Results and Discussion

### 2.1 Design, Fabrication, and Characterization of the HEC‐TENG

The deformable and large‐area HEC‐TENG developed in this study exhibited excellent applicability in practical scenarios, as shown in Figure 1a. It not only harvests droplet energy under rainy conditions but can also be directly integrated into clothing to collect contact/noncontact biomechanical energy. As illustrated in Figure 1b, the entire HEC‐TENG has a three‐layered architecture. The SR layer doped with HEC particles serves as the triboelectric charge trapping and transport layer, the middle GT functions as the charge storage layer, and the bottom SR layer blended with CB serves as a stretchable electrode. To prepare the HEC particles, all precursor materials (ZrCl_4_, SnCl_2_, TiO_2_, SrCl_2_·6H_2_O, HfCl_4_, and NbCl_5_) were first mixed into a homogeneous suspension and then subjected to a hydrothermal reaction at 200°C for 9 h, yielding Sr(Ti_0.2_Sn_0.2_Zr_0.2_Hf_0.2_Nb_0.2_)O_3_ (STSZHN) particles (Figure 1c, i). Compared with conventional high‐pressure solid‐state sintering, this method markedly simplifies the synthesis process. The cotton textile was carbonized at 1000°C for 12 h to form GT, which served as a triboelectric charge storage layer (Figure 1c, ii). Subsequently, a three‐layered HEC‐TENG was fabricated via sequential encapsulation in the SR. Briefly, HEC particles were uniformly dispersed in SR and cast into a mold with sandpaper‐induced microstructures at the bottom to form a triboelectric charge trapping/transport layer. After curing, the GTs were placed on the HEC‐SR layer and embedded in the SR to construct the middle layer. Finally, the CB/SR composite was coated as a stretchable electrode layer (Figure 1c, iii).

**FIGURE 1:**
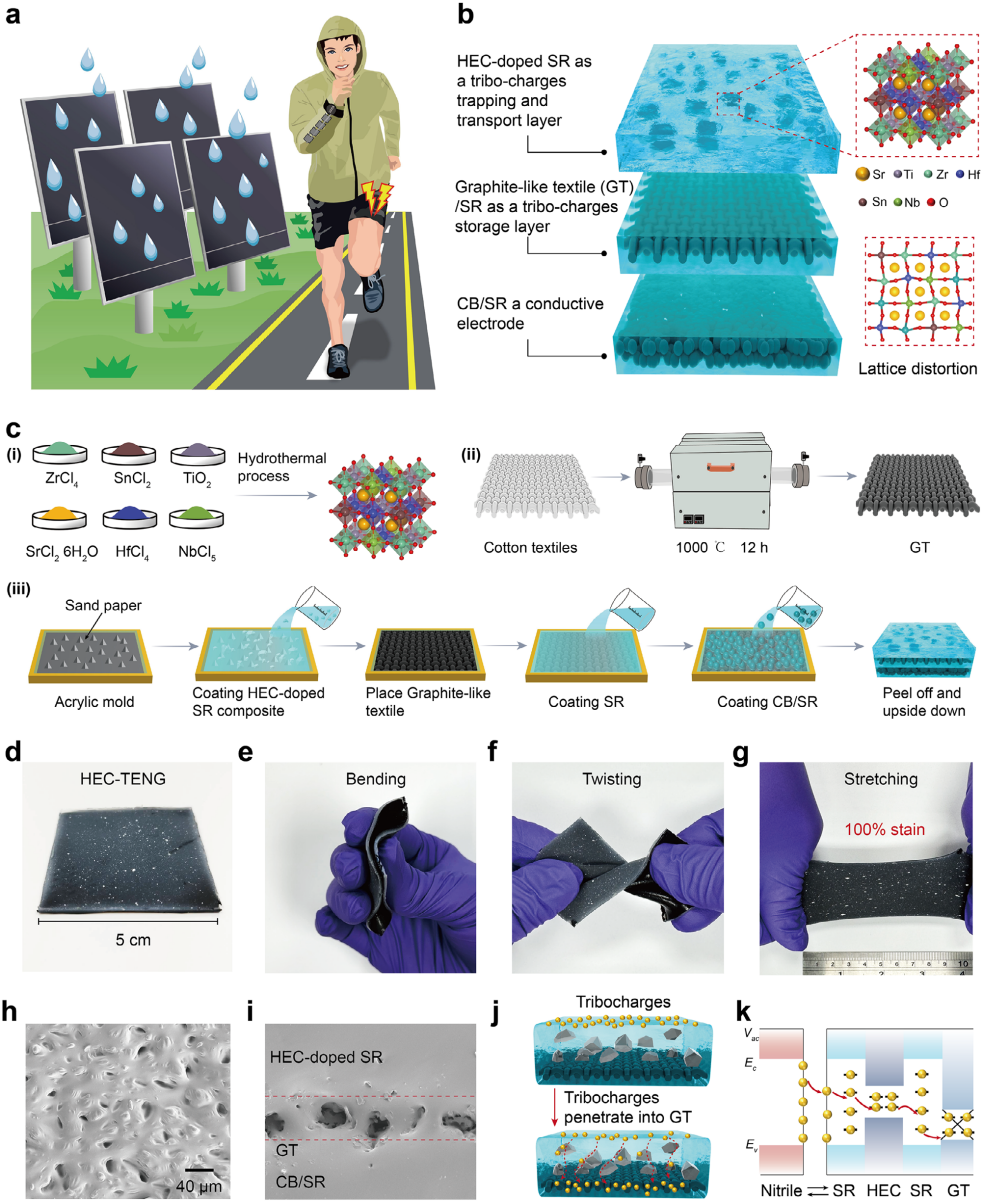
Schematic of deformable HEC‐TENGs. (a) Schematic of wearable noncontact biomechanical energy harvesting, droplet energy scavenging, and rain power panels enabled by HEC‐TENGs. (b) Exploded view of the HEC‐TENG configuration, including a schematic of the HEC crystal structure and its lattice distortion. (c) Fabrication process of HEC‐TENG, including (i) HEC particles synthesized via hydrothermal process, (ii) GT carbonized from cotton textile, (iii) construction process of tri‐layered HEC‐TENG. (d) Photographs of HEC‐TENG (5 × 5 cm^2^). (e–g) Photographs of deformable HEC‐TENG under bending, twisting, and stretching. (h) Top‐view scanning electron microscope (SEM) image of HEC‐TENG, showing the coarse surface. (i) Cross‐sectional SEM image of HEC‐TENG device, revealing the triple‐layered structure. (j) Schematic of tribo‐charges penetration into GT. (k) Band diagram of triboelectric charge transfer in HEC‐TENG.

Upon complete curing of SR, a soft and deformable HEC‐TENG (5 × 5 cm^2^) was obtained (Figure 1d). As SR encapsulated all three layers of the HEC‐TENG, the device could withstand various deformations and stretching, including folding, twisting, and 100% strain (Figure 1e–g). Notably, the entire HEC‐TENG can withstand strains as high as 230% (Figure S1). As shown in Figure 1h, the top‐view scanning electron microscope (SEM) image reveals a rough surface peeled off from the sandpaper mold (Figure S2). The cross‐sectional SEM images in Figures 1i and S3 confirm that the HEC particles, GT, and CB, are uniformly embedded within the SR matrix, forming a well‐defined multilayered architecture. With a multilayer design, the HEC‐TENG operates via the following mechanism: triboelectric charges are generated on its surface upon contact with external objects. These charges then penetrate the GT via the HEC particles, which not only trap the charges for short‐term storage but also enhance the dielectric performance. Subsequently, the GT, serves as a charge‐storage layer, thereby prolongs charge retention (Figure 1j). The corresponding energy band diagram of the working system is shown in Figure 1k.

Detailed material characterization of the HEC particles and GT is shown in Figure 2. The X‐ray diffraction (XRD) pattern of the STSZHN particles shows that the cubic SrTiO_3_‐type typical peaks ((100), (110), (111), (200), (211), (220), (310)) are retained without including impurity peaks, indicating a single‐phase solid solution (Figure 2a). All diffraction peaks are systematically shifted toward lower 2θ angles relative to the SrTiO_3_ reference (PDF 35‐0734), suggesting an expansion of the lattice with an estimated a ≈ 4.087 Å. This lattice expansion can be rationalized by incorporating larger B‐site cations, Zr^4+^ (0.72 Å), Hf^4+^ (0.71 Å), Sn^4+^ (0.69 Å), and Nb^5+^ (0.64 Å), in place of Ti^4+^ (0.605 Å) [35], which collectively increase the average ionic radius at the B‐site, thus enlarging the unit cell. Figure 2b displays a high‐resolution transmission electron microscopy (HR‐TEM) image of the densely agglomerated nanocrystals with clearly resolved lattice fringes. The inset presents the corresponding fast Fourier transform pattern obtained from the selected area (white circle), which exhibits well‐defined diffraction spots characteristic of a single‐crystalline domain oriented along the [111] zone axis.

**FIGURE 2:**
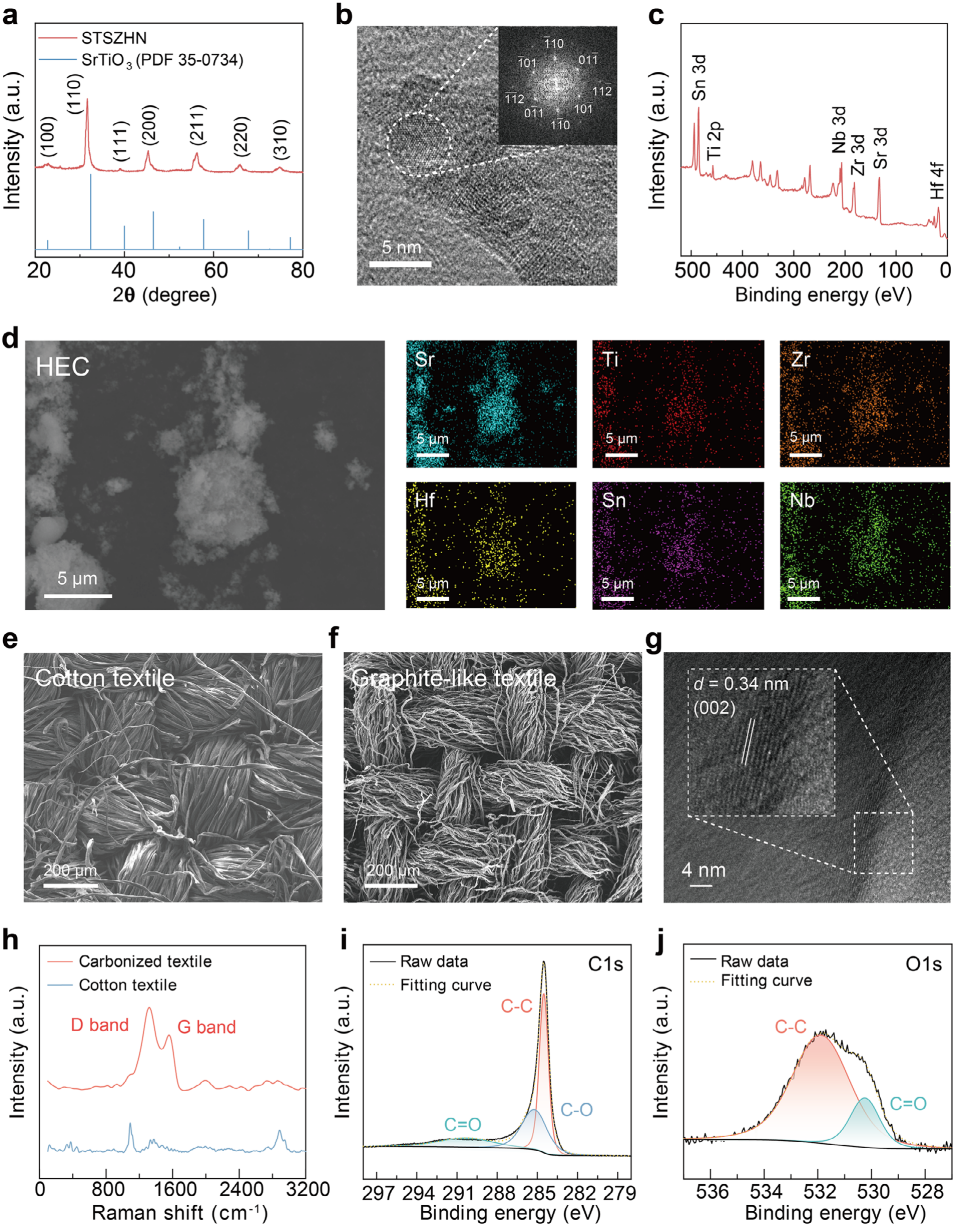
Materials characterization of HEC and GT. (a) XRD pattern of HEC particles. (b) HR‐TEM image and corresponding selected area electron diffraction pattern of HEC particles. (c) XPS spectra of HEC particles. (d) EDS elemental mapping of HEC showing Sr, Ti, Zr, Hf, Sn, and Nb. (e) SEM images of pristine cotton textile and (f) GT. (g) HR‐TEM image of GT. (h) Raman spectra of cotton textile and carbonized textile. (i–j) High‐resolution XPS spectra of GT.

Figure 2c shows the survey X‐ray photoelectron spectrum (XPS) of the HEC particles, which clearly shows distinct core‐level signals for Sr, Ti, Zr, Hf, Sn, and Nb, confirming the multicationic composition of the sample. The detailed valence states of these cations were analyzed in the corresponding high‐resolution spectra (Figure S4). For Sr, the 3d spectrum can be deconvoluted into two characteristic doublets: bulk components at 132.6 eV and 134.3 eV (Sr 3d_5/2_ and Sr 3d_3/2_) and surface components at 134.3 eV and 136.0 eV, a typical feature of Sr‐based perovskite oxides (Figure S4a) [36, 37]. As shown in Figure S4b,c, the Ti 2p peaks at 457.9 eV (2p_3/2_) and 463.6 eV (2p_1/2_) correspond to the Ti^4+^ oxidation state [38, 39]. Whereas the Zr 3d doublet located at 181.6 eV and 183.9 eV can be assigned to Zr^4+^ 3d_5/2_ and 3d_3/2_, respectively [40, 41]. The Hf 4f spectrum displays a more complex profile, which can be fitted into two oxidation states: Hf^3+^ (4f_7/2_ at 16.0 eV and 4f_5/2_ at 17.7 eV) and Hf^4+^ (4f_7/2_ at 18.0 eV and 4f_5/2_ at 19.8 eV) [42, 43]. A nearby Sn 4d doublet (4d_5/2_ at 25.3 eV and 4d_3/2_ at 26.4 eV) was also observed at higher binding energies [43, 44]. Consistently, the Sn 3d peaks at 486.1 eV and 494.4 eV correspond to Sn^4+^ 3d_5/2_ and 3d_3/2_, indicating the absence of other oxidation states (Figure S4e) [45]. In the Nb 3d spectrum (Figure S4f), the peaks centered at 206.6 eV and 209.6 eV are attributed to Nb^5+^ 3d_5/2_ and 3d_3/2_, respectively [43, 44, 46, 47]. Additionally, a broadened feature at higher binding energies can be ascribed to the Hf 4d_5/2_ orbitals, which comprise contributions from Hf^3+^ (211.1 eV) and Hf^4+^ (213.1 eV) [48]. These XPS results confirm that the as‐synthesized HEC particles formed a homogeneous multicationic perovskite solid solution with well‐defined cationic valence distributions. Furthermore, SEM and energy‐dispersive X‐ray spectroscopy (EDS) images revealed that the HEC particles were dispersed throughout the SR matrix, confirming the presence of Sr, Ti, Zr, Hf, Sn, and Nb, which is consistent with the XPS results (Figure 2d).

As reported in our previous work, GT, with its hierarchical interwoven architecture with abundant fiber‐fiber interfaces, provides a large effective contact area and is used as a triboelectric charge storage layer [3, 23]. We subsequently conducted analogous materials characterizations on cotton textile and carbonized GT. Although carbonization partially disrupted the interwoven architecture of the GT relative to the pristine cotton textile, abundant fibrous features were preserved (Figure 2e–f). The corresponding EDS analysis is presented in Figure S5. In addition, Figure 2g shows an HR‐TEM image of the GT, revealing lattice fringes with a spacing of approximately 0.34 nm corresponding to the (002) planes [49]. Figure 2h shows the Raman spectra of the cotton textile and GT. Relative to cotton, GT exhibits a D band at 1318 cm^−^^1^ and a G band at 1555 cm^−^^1^. A weak and wide band appears at approximately 2750 cm^−^^1^, indicating the possible disordered graphitic domains or heteroatom incorporation. Figure 2i,j show the elemental composition and chemical bonding of GT using high‐resolution XPS. The C 1s spectrum is deconvoluted into peaks at 284.5 eV (sp^2^ C─C/C═C), 285.2 eV (C─O), and 290.5 eV (C═O), indicating a predominantly graphitic framework with a small fraction of oxygenated functionalities [23, 50]. Consistently, the O 1s region is dominated by a C─C component with a minor C═O contribution.

### 2.2 Configuration Design, Mechanism and Optimization of the HEC‐TENG

To investigate the effects of the HEC particle doping concentration and device configuration on the output performance of the TENG, a single‐electrode contact‐separation mode was employed to generate electrical signals. Figure 3a illustrates the working principle of the HEC‐TENG operating in the contact‐separation mode. Herein, the HEC‐doped SR and nitrile were employed as the two triboelectric layers. When the two layers are in contact, the HEC‐doped SR layer tends to gain electrons owing to the triboelectrification effect. It exhibits a negative surface potential as it exhibits a stronger electronegativity than the nitrile. The presence of the HEC particles facilitated charge trapping/transfer toward the underlying GT layer, where the transferred charges could be effectively redistributed and stored. During the separation process, positive charges flowed from the ground to the CB electrode owing to the electrostatic induction effect, generating an electric field at the GT/CB interface to maintain charge neutrality and produce current through the external circuit. When the two triboelectric layers reached maximum separation distance, the induced charges on the CB electrode reached their peak value, resulting in the highest output signal in the external circuit. Subsequently, during the approach process of the two triboelectric layers, the positive charges on the nitrile partially neutralized the negative charges at the HEC‐doped SR interface, thereby weakening the electrostatic induction effect at the CB electrode. Consequently, the previously accumulated positive charges flowed to the ground, generating an opposite current in the external circuit. Once the two layers were in full contact, complete charge neutralization occurred between the HEC‐doped SR and nitrile layers, and the positive charges on the CB electrode were fully released, leading to no current output in the external circuit. During the cyclic contact‐separation process, alternating pulse‐like electrical signals were generated in the external circuit.

**FIGURE 3:**
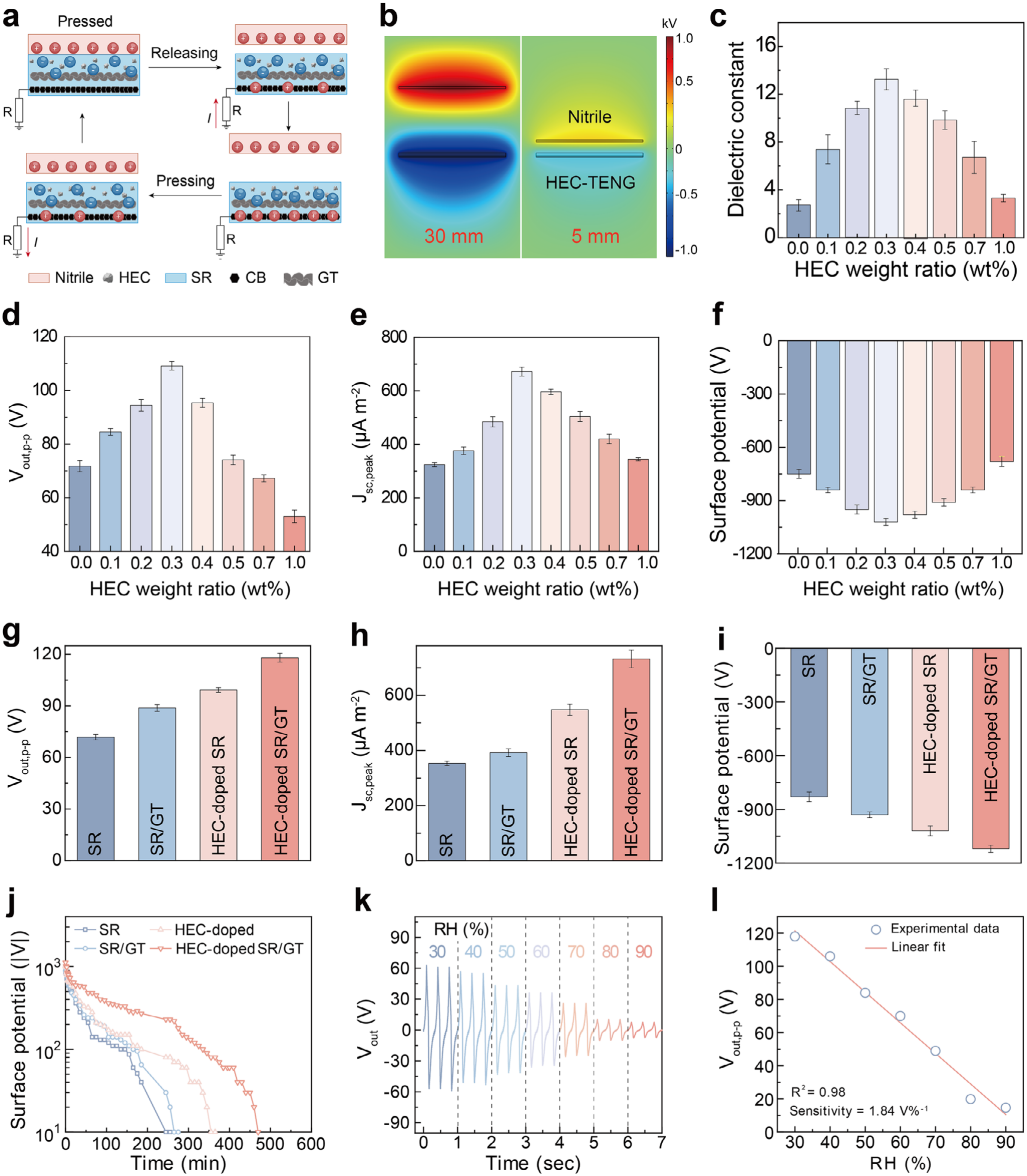
Working mechanism and performance optimization of HEC‐TENG in contact‐separation mode. (a) Schematic of working mechanism in contact‐separation mode. (b) Simulated potential distribution during the approach and separation processes obtained from COMSOL. (c) Dielectric constant of HEC‐doped SR at different HEC weight ratios (0–1 wt%). Testing frequency: 1 kHz. (d) V_out, p‐p_, (e) J_sc, peak_, and (f) surface potential of HEC‐TENG with different HEC weight ratios (0–1 wt%). (g) V_out, p‐p_, (h) J_sc, peak_ and (i) surface potential comparison among devices fabricated by SR, SR/GT, HEC‐doped SR, and HEC‐doped SR/GT. (j) Variation of surface potential with time for different configurations (including SR, SR/GT, HEC‐doped SR, and HEC‐doped SR/GT. (k) V_out_ under different RHs (35%–75%). (l) Corresponding linear fitting curve.

As shown in Figure 3b, COMSOL simulations were conducted to visualize the variation in the electric field near the two triboelectric layers during the separation and approach processes under open‐circuit conditions. The electric field intensity increased with a significant separation distance (30 mm). It decreased as the two layers approached each other (5 mm), thereby driving the current variation in the external circuit. The transferred charge density is positively correlated with the dielectric constant of the triboelectric layer, which directly determines the electrical output of the TENG [3, 23, 27, 51, 52, 53]. Furthermore, the quantitative relationship between the transferred charge density and the dielectric constant can be expressed as follows:

(1) $$\sigma^{\prime }=\frac{\sigma_{0}d_{gap}}{d_{gap}+d/\varepsilon }\;$$

where 𝜎′ and 𝜎_0_ are the maximum transferred charge density and the pristine triboelectric charge density (at the equilibrium state), respectively; d_gap_ is the gap distance; d and ε are the dielectric constant and thickness of the triboelectric layer, respectively. Based on this, we first investigated the variation in the dielectric constant of the HEC‐doped SR layer with different HEC doping concentrations (Figure 3c). When the HEC doping concentration reached 0.3 wt%, the dielectric constant reached a maximum value of 13.25, attributed to the formation of numerous microcapacitors that effectively retain triboelectric charges [3, 23]. By comparison, when other ceramic particles, including Al_2_O_3_, BaTiO_3_, and TiO_2_, were incorporated at the same loading ratio of 0.3 wt%, no significant increase in the dielectric constant was observed (Figure S6). However, with a further increase in HEC doping concentration, the dielectric constant gradually decreased, which may be attributed to the formation of conductive pathways within the SR matrix at excessive HEC loading, leading to the percolation and dissipation of triboelectric charges. In addition, local agglomeration occurs at high HEC particle loading ratios (>0.5 wt%), reducing the effective filler‐matrix interfacial area and disrupting homogeneous interfacial polarization (Figure S7a). Furthermore, the Young's modulus, fractured stress, and strain of the HEC‐doped SR remained nearly unchanged over the 0–1 wt% loading range, suggesting that mechanical compliance is unlikely to be a main factor affecting the TENG output performance (Figure S7b).

Subsequently, the output performance of the HEC‐doped SR/GT TENGs (5 × 5 cm^2^, 3 mm thickness) with different HEC doping ratios was systematically measured and compared at an operating frequency of 3 Hz and a contact force of 15 N. As the HEC weight ratio increased from 0 to 1 wt%, the peak‐to‐peak output voltage (V_out, p‐p_) exhibited an initial increase followed by a decrease. The maximum V_out, p‐p_ of 110 V was obtained at an HEC concentration of 0.3 wt% (Figure 3d). The corresponding time‐dependent output voltage (V_out_) curves are presented in Figure S8a. Furthermore, peak short‐circuit current density (J_sc, peak_) of the HEC‐TENG exhibited a similar trend, reaching a maximum value of 640 µA m^−^^2^ at an HEC concentration of 0.3 wt% (Figure 3e). The corresponding time‐dependent J_sc_ curves are presented in Figure S8b. In addition, V_oc_ and transferred charge (Q_tr_) were measured using a Keithley 6514 electrometer. Similar variation trends were observed in the V_oc_ and Q_tr_ output curves, reaching a maximum V_oc_ of 90 V and a maximum Q_tr_ of −49 nC at an HEC concentration of 0.3 wt% (Figure S9a,b). Surface potential measurements further confirmed that incorporating HEC particles into the SR matrix significantly enhanced the surface potential, reaching a maximum of −1020 V at an HEC concentration of 0.3 wt% (Figure 3f). The results indicate that incorporating HEC particles into the SR triboelectric layer markedly improved the V_oc_, J_sc_, and Q_tr_ outputs of the TENG, reaching a maximum performance at an HEC concentration of 0.3 wt%. However, a further increase in HEC content led to the formation of conductive percolation paths and local agglomeration, resulting in charge dissipation and reduced output performance [25, 33, 34]. Therefore, an HEC doping concentration of 0.3 wt% was identified as the optimal ratio for subsequent experimental studies. Notably, when 0.3 wt% of Al_2_O_3_, BaTiO_3_, or TiO_2_ ceramic particles was incorporated into the SR matrix, the TENG output performance showed little improvement, which is consistent with the corresponding dielectric constant trends (Figure S10a–d).

Compared to contact‐separation TENGs, noncontact TENGs require stronger charge‐trapping and charge‐retention capabilities to achieve a high triboelectric output. In addition to incorporating HEC particles into the SR layer, a GT layer was introduced as a charge reservoir to enhance charge storage. To verify that the fabricated three‐layer composite HEC‐TENG exhibited the highest output performance, four types of TENGs with different triboelectric layer configurations were prepared, pure SR, SR/GT bilayer, HEC‐doped SR, and HEC‐doped SR/GT bilayer. Their output performance was evaluated under the same testing conditions (3 Hz and 15 N). As shown in Figures 3g,h and S11a,b, the HEC‐doped SR/GT TENG exhibited the highest V_out, p‐p_ and J_sc, peak_ among the four configurations, with maximum values of 118 V and 732 µA m^−2^. In comparison, the pure SR‐based TENG delivered only 72 V and 353 µA m^−2^. For the SR‐based triboelectric layer, the introduction of a GT layer and the incorporation of HEC particles enhance the triboelectric charge storage and charge trapping capabilities of the TENG, respectively. By integrating these two effects, the HEC‐doped SR/GT design simultaneously achieves high charge trapping and storage capacities, leading to the highest TENG output. The corresponding time‐dependent V_out_ and J_sc_ curves are presented in Figure S11a,b. Similarly, the HEC‐doped SR/GT TENG exhibited the highest V_oc_ of 102 V and Q_tr_ of ‐59 nC (Figure S12a,b). Furthermore, Surface potential measurements also confirmed that the HEC‐doped SR/GT triboelectric layer exhibited the highest surface potential of −1120 V (Figure 3i). As shown in Figure 3j, the surface potential retention time of the HEC‐doped SR/GT triboelectric layer reached 470 min, which was markedly higher than those of the HEC‐doped SR (355 min), SR/GT (275 min), and pure SR (245 min) layers. These results demonstrate that the HEC‐doped SR/GT configuration enables efficient capture and storage of triboelectric charges, with prolonged charge retention capability.

To evaluate the applicability of the HEC‐TENG under harsh environmental conditions, its output performance was investigated at different relative humidity (RH) and temperatures. Figure 3k,l, as the RH increased from 30% to 90%, the V_oc_ decreased from 118 V to 14.7 V, exhibiting a linear correlation with RH variation, with a sensitivity of 1.84 V %^−^^1^. Similarly, the variation curves and linear fitting relationships of V_oc_, J_sc_, and Q_tr_ for RH levels ranging from 30% to 90% are shown in Figure S13a–f. The decrease in the HEC‐TENG performance with increasing RH is mainly attributed to the adsorption of water molecules on the triboelectric surfaces, which enhances the surface conductivity and accelerates charge dissipation. The variation curves and linear fitting relationships of V_oc_, J_sc_, and Q_tr_ for the HEC‐TENG within the temperature range of 303–343 K are presented in Figure S14a–f. The decline in HEC‐TENG output with increasing temperature was mainly owing to enhanced charge leakage and reduced charge retention. These results indicate that the HEC‐TENG exhibited a sensitive response to external humidity and temperature variations, suggesting its potential as a flexible, self‐powered humidity and temperature sensor.

### 2.3 Performance of HEC‐TENG for Contact/Noncontact Energy Harvesting

We systematically evaluated the output performance of the optimized HEC‐TENG device in both contact and noncontact operating modes. The device has dimensions of 5 × 5 cm^2^ with a thickness of 3 mm. First, in the contact‐separation mode, the HEC‐TENG was operated at a constant contact pressure of 15 N, whereas the contact frequency was changed from 1 to 4 Hz. The corresponding V_out_ and J_sc_ outputs are shown in Figure 4a,b. As the contact frequency increased from 1 to 4 Hz, the V_out_ and J_sc_ outputs increased from 43 to 140 V and from 0.3 to 1.0 mA m^−^^2^, respectively. In contrast, the outputs of V_oc_ and Q_tr_ remained nearly unchanged as the contact frequency increased from 1 to 4 Hz, maintaining values of 100 V and ‐60 nC, respectively (Figure S15a,b). When the applied force was varied, both V_out_ and J_sc_ increased linearly. This trend can be attributed to the enlarged effective contact area between the two triboelectric layers under the contact force, which facilitates greater charge transfer and thus enhances the electric output. Specifically, within the 0–4 N range, the linear sensitivities of V_out, p‐p_ and J_sc, peak_ reached 23.65 V N^−1^ and 0.25 mA m^−2^ N^−1^, respectively, while in the 4–10 N range, the corresponding sensitivities were 2.71 V N^−1^ and 0.03 mA m^−2^ N^−1^, respectively (Figure S16). The response time and long‐term output stability under cyclic pressure are presented in Figure S17a,b. Subsequently, to evaluate the maximum power density output of the HEC‐TENG, the device was connected to external load resistors with various resistances. The corresponding voltage and current density outputs were recorded under each condition. As shown in Figure 4c,d, voltage gradually increased with increasing external resistance and reached saturation at approximately 5 MΩ, whereas current density decreased with increasing external resistance. Under different resistive loads, the power density was calculated by multiplying the load voltage and current density. At a contact frequency of 4 Hz, the maximum power density achieved 35.7 mW m^−^^2^, corresponding to an optimized external resistance of 44 MΩ (Figure 4e). At lower frequencies of 1, 2, and 3 Hz, the maximum power density values were 13.5, 19.5, and 32.5 mW m^−^^2^, respectively. The long‐term operational stability of the HEC‐TENG was subsequently evaluated, as shown in Figures 4f, S18a, and S18b. During 5000 continuous operation cycles, the outputs of V_oc_, J_sc_, and Q_tr_ remained nearly unchanged, showing no noticeable degradation in performance. This result confirms the excellent long‐term stability and durability of HEC‐TENG during repeated operations. Furthermore, HEC‐TENG exhibited robust V_out_ when in contact with a variety of triboelectric materials (Figure S19).

**FIGURE 4:**
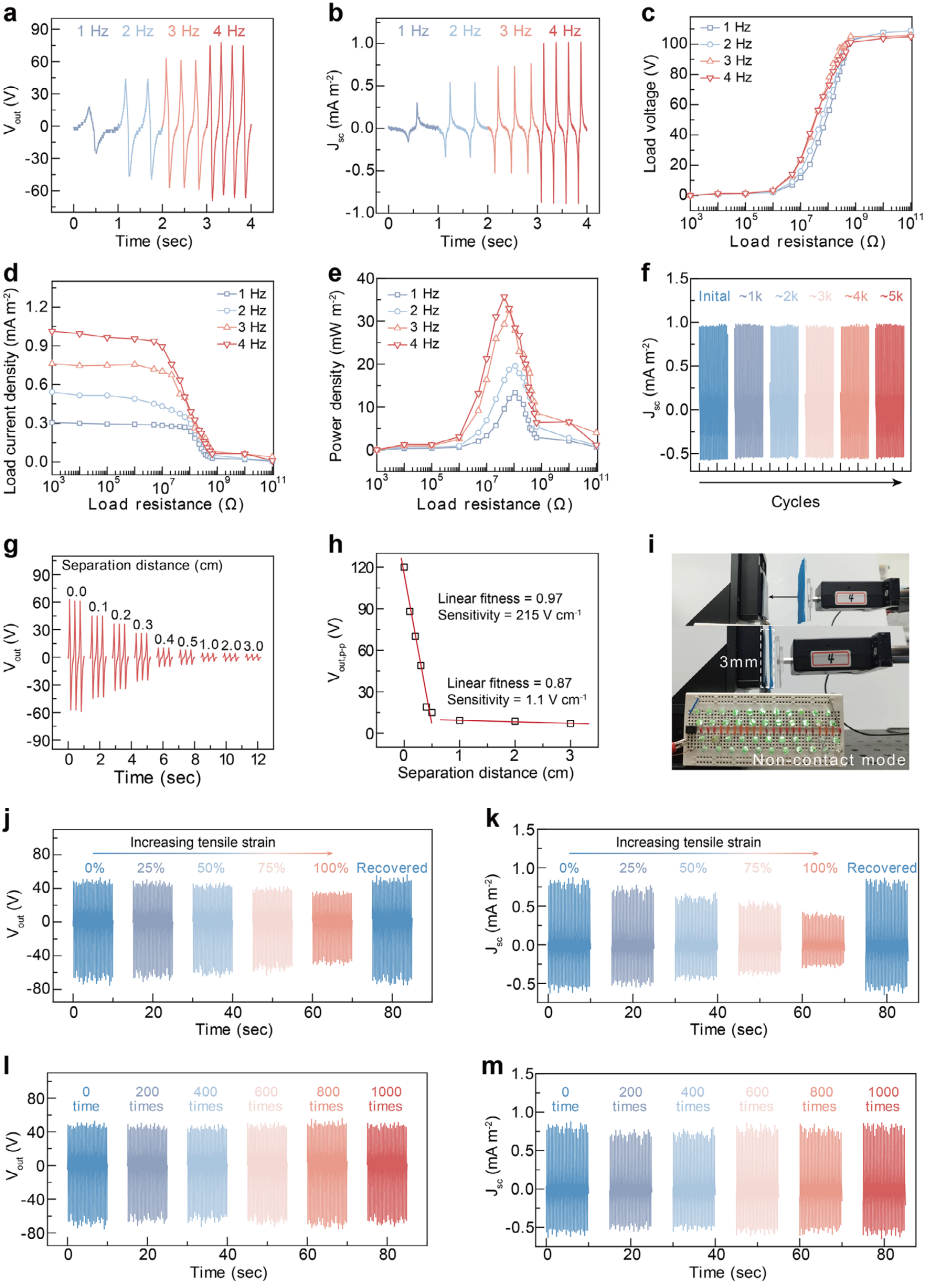
Output performance of HEC‐TENG. (a) V_out_ and (b) J_sc_ performance output under different contact frequencies (1–4 Hz). (c) Voltage, (d) Current density, and (e) Power density at different load resistances under different contact frequencies (1–4 Hz). (f) J_sc_ in continuous operation from 1 to over 5000 cycles. (g) V_out_ of noncontact HEC‐TENG with different separation distances from 0–3 cm. (h) Relationship between V_out, p‐p_ and separation distance fitting by a linear curve. (i) Photograph of LEDs lit by HEC‐TENG at a 3 mm separation distance. (j) V_out_ and (k) J_sc_ under different tensile strains (0–100 %). (l) V_out_ and (m) J_sc_ under 100% tensile strain measured over 1000 cycles.

Next, the output performance of the HEC‐TENG in noncontact mode was evaluated. Prior to the noncontact measurements, the HEC‐TENG was operated under the contact–separation mode until a stable output was achieved, allowing triboelectric charges to be pre‐generated on the HEC‐doped SR surface and subsequently stored in the GT layer. Subsequently, noncontact TENG measurements were conducted at a preset separation distance, and the corresponding working mechanism is illustrated in Figure S20. The V_out_ of the HEC‐TENG at different separation distances (0–3 cm) in the noncontact mode is shown in Figure 4g. As the separation distance increased from 0 to 3 cm, the V_out, p‐p_ gradually decreased from 120 V to 7.2 V. The linear fitting relationship between the V_out, p‐p_ and the separation distance is illustrated in Figure 4h. Within the range of 0–0.5 cm, the V_out_ exhibited the highest sensitivity of 215 V cm^−^^1^, whereas at larger distances of 0.5–3 cm, the sensitivity decreased to 1.1 V cm^−^^1^. The changes in V_oc_, J_sc_, and Q_tr_ with respect to the separation distance are presented in Figure S21a–c, exhibiting similar trends with that observed from the V_oc_ variations. Notably, the J_sc, peak_ values at separation distances of 0.1, 0.5, and 1 cm were 450, 120, and 30 µA cm^−^^2^, respectively. These values are higher than those of many recently reported noncontact TENGs (Table S1) [27, 53, 54, 55, 56, 57, 58, 59, 60]. In addition, at the separation distance of 0.3 cm, the V_oc_ output remained nearly unchanged over 6000 operation cycles (Figure S22). Under this condition, the HEC‐TENG was also capable of lighting multiple LEDs, further demonstrating its promising practical applicability (Figure 4i).

To verify the operational stability of the HEC‐TENG during mechanical stretching and deformation, the output performance was evaluated under various tensile strains. The tensile strain was calculated using the following equation:

(2) $$\varepsilon =\frac{L-L_{0}}{L_{0}}\times 100\%\;\;\;$$

where L and L_0_ represent the stretched and initial lengths of the device, respectively. When the tensile strain increased from 0% to 100%, the HEC‐TENG exhibited only a slight decrease in output performance: the V_out, p‐p_ decreased from 112 V to 85 V, and the peak‐to‐peak J_sc_ values decreased from 1.34 mA m^−^^2^ to 0.72 mA m^−^^2^ (Figure 4j,k). This partial performance loss can be attributed to the increased resistance of the CB/SR electrode under tensile strain, which weakens electrostatic induction and reduces the triboelectric output. Furthermore, under cyclic stretching at 100% strain for 1000 cycles, the V_out_ and J_sc_ outputs measured after different cycles are shown in Figure 4l,m. Both the V_out_ and J_sc_ outputs remained highly stable throughout the cyclic stretching tests. These results confirm that the HEC‐TENG maintained robust, durable triboelectric output under various deformation and stretching conditions.

### 2.4 Performance Output of HEC‐TENG for Droplet Energy Harvesting

Owing to the excellent hydrophobicity and strong dielectric properties of the HEC‐doped SR layer, the HEC‐TENG also exhibited a good droplet‐based energy generation capability. Figure 5a illustrates the working mechanism of droplet‐based electricity generation. Specifically, as the droplet falls onto the surface of the HEC‐doped SR layer, the inherent electronegativity of SR combined with the strong dielectric nature of HEC induces the accumulation of negative charges on the SR surface. Simultaneously, some negative charges are transferred toward the GT layer with the assistance of the HEC particles, leading to the induction of positive charges on the CB layer. Once the droplet contacts the HEC‐doped SR surface, positive charges accumulate in the droplet close to the negatively charged HEC‐doped SR surface owing to electrostatic induction, whereas negative charges accumulate in the region distant from the surface. After the droplet impacts the hydrophobic SR surface, it continues to spread outward, enlarging the liquid‐solid interfacial area and thereby enhancing the EDL effect. When the droplet spreads on the negatively charged HEC‐doped SR surface and contacts the top electrode, the droplet/top electrode connection forms a transient conductive path. Electrons are driven from the top electrode toward the bottom CB/SR electrode through the external circuit, leaving the top electrode positively charged. During droplet contraction and detachment, the interfacial EDL area decreases, and the charge distribution relaxes, resulting in a reverse current. When the droplets are applied continuously, the process is repeated periodically, resulting in a series of continuous peak current outputs. Furthermore, since no corona discharge phenomena were observed during the droplet energy‐harvesting process, it can be confirmed that the peak voltage and current outputs do not originate from electrostatic breakdown (Figure S23a,b) [61].

**FIGURE 5:**
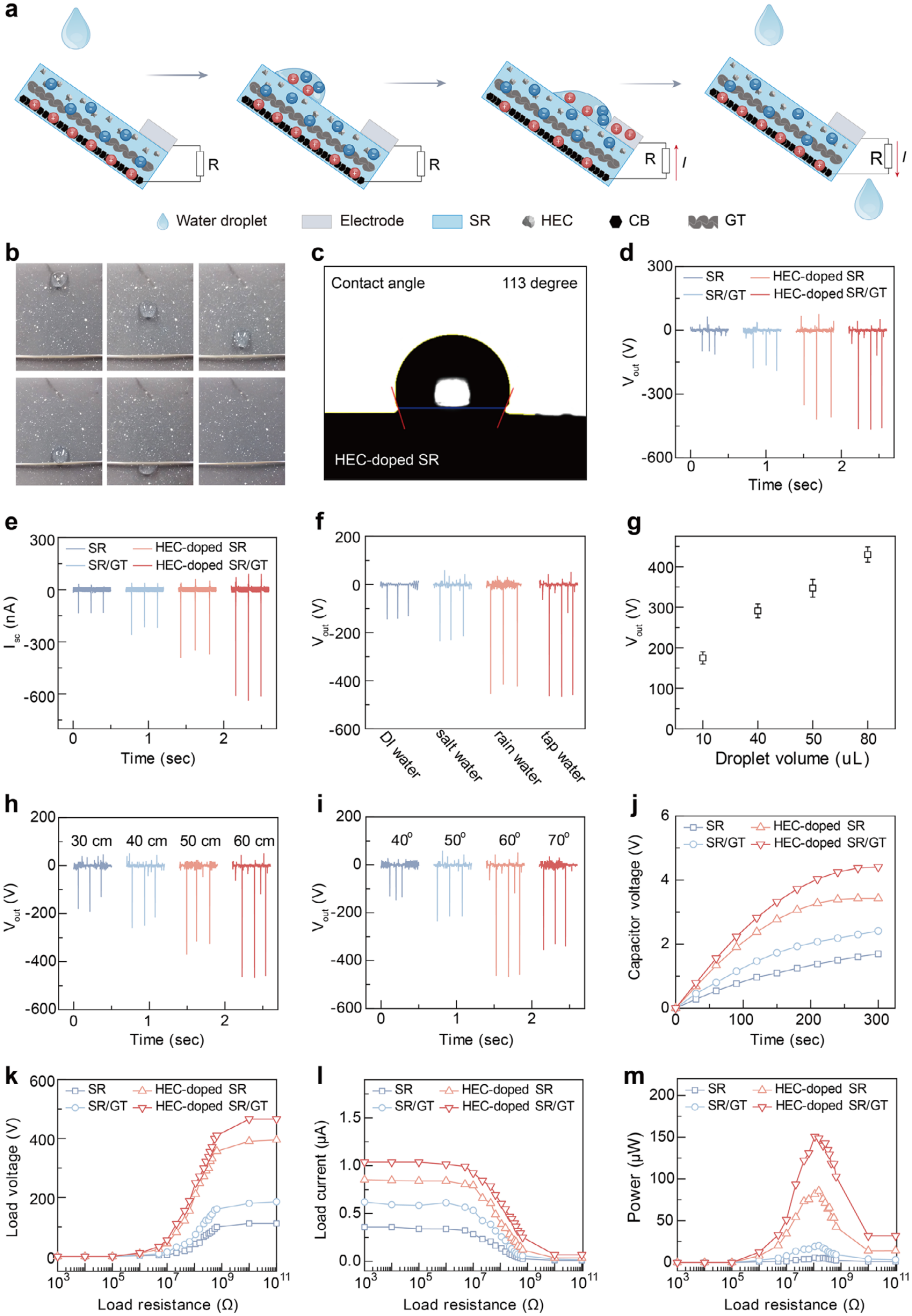
Output performance of HEC‐TENG in liquid‐solid contact mode. (a) Working mechanism. (b) Photographs of water droplets sliding off the top surface of HEC‐TENG. (c) Contact angle of a water droplet on the surface of HEC‐doped SR. (d) V_out_ and (e) I_sc_ of HEC‐TENGs with different configurations (including SR, SR/GT, HEC‐doped SR, and HEC‐doped SR/GT). (f) V_out_ with different water droplet sources. (g) V_out_ among different droplet volumes (10–80 µL). (h–i) V_out_ of HEC‐TENG with different water droplet tilt angles and height. (j) Charging curves of a capacitor with time variation of different configurations. (k) Voltage, (l) Current, and (m) Power at different resistance loads under different configurations.

The HEC‐doped SR surface exhibited a contact angle of 113°, indicating excellent hydrophobicity. Moreover, throughout the droplet movement cycle, including impact, contact with the SR surface, interaction with the top electrode, and detachment, no water residue was observed on the surface (Figure 5b,c). This demonstrates that droplet‐based energy generation can effectively avoid interfacial wear associated with contact‐separation friction, thereby enabling long‐term stable operation. Subsequently, the droplet‐based energy generation performance of the HEC‐TENG was compared for different device configurations (Figure 5d,e). Among the SR, HEC‐doped SR, and SR/GT configurations, the HEC‐doped SR/GT configuration exhibited the highest output performance, achieving a V_out_ of 466 V and I_sc_ of 637 nA. In contrast, the single‐layer SR configuration generated a V_out_ of 112 V and an I_sc_ of 134 nA. These results demonstrate that both the incorporation of HEC particles and the introduction of the GT interlayer enhance the DEG output. In addition, factors such as the ionic concentration, volume, and falling height of the droplets, as well as the tilt angle of the device, influence the droplet‐based energy harvesting performance of the HEC‐TENG. Compared with deionized (DI) water, saline solution, rainwater, and tap water exhibited enhanced V_oc_ outputs (Figure 5f). This enhancement can be attributed to the presence of trace salt ions, which reduce the Debye length of the EDL and increase the ionic conductivity of the droplets [6, 62]. As the droplet volume increased from 10 µL to 80 µL, the V_out_, I_sc_, and Q_tr_ all exhibited a linear increase (Figures 5g and S24a,b). This can be attributed to the increased spreading area of the droplet, which expands the effective EDL area and further enhances the electricity generation output.

Furthermore, the falling height of the droplet affected the output of HEC‐TENG. As the height increased from 30 to 60 cm, both V_out_ and I_sc_ showed a linear increase (Figures 5h and S25a), which can be attributed to the enlarged spreading area of the droplet upon impact at higher falling heights. As shown in Figures 5i and S25b, V_out_ and I_sc_ reached their maximum values when the device was tilted at 60°, indicating that this inclination provides the largest droplet spreading area upon impact. Subsequently, we demonstrated that HEC‐TENG could charge capacitors under all four device configurations, with the HEC‐doped SR/GT configuration exhibiting the most pronounced charging curve (Figure 5j). When connected to external load resistors with varying resistance values, the corresponding voltage and current outputs were measured, as shown in Figure 5k,l. After calculation, the HEC‐doped SR/GT configuration achieved a maximum power output of 150 µW at an external resistance of 110 MΩ (Figure 5m). For comparison, the pure SR configuration generated only 5.78 µW of power output under the same external resistance of 110 MΩ.

A detailed comparison of recently reported TENGs with noncontact energy or droplet‐based energy harvesting is provided in Table S1. In contrast to previous studies that only demonstrated noncontact or liquid‐solid contact outputs, our designed HEC‐TENG enables simultaneous energy harvesting in three operation modes, including contact‐separation, noncontact, and liquid‐solid contact modes, and exhibits outstanding output performance. Moreover, the tri‐layer composite configuration (HEC‐doped SR/GT/CB) endows the device with exceptional overall stretchability (up to 230% strain), suggesting high potential for practical wearable and distributed energy applications.

### 2.5 Applications of HEC‐TENG

Previously reported contact‐separation‐mode stretchable TENGs integrated beneath clothing are inefficient at harvesting mechanical energy from subtle body motions [23]. This is because efficient TENG operation requires continuous and seamless contact‐separation processes, whereas in practical situations, the body does not continuously contact the clothing during motion. As a result, a considerable portion of biomechanical energy cannot be effectively converted into electrical output. Therefore, such contact‐separation‐type TENGs are generally applicable only to specific body regions, particularly joints such as the knees (Figure S26). In addition, the long‐term contact and separation between SR elastomers and skin may induce allergic reactions and bacterial infections. In contrast, owing to its excellent noncontact TENG performance, combined with fabric‐like softness and full stretchability, our HEC‐TENG can effectively overcome the above limitations. It can be integrated into clothing over large areas without being restricted to specific body locations, thereby broadly harvesting biomechanical energy.

As shown in Figure 6a–d, when integrated on the inner surface of a short‐sleeved top and shorts, the HEC‐TENG can light several LEDs while hand swinging, running, and jumping without physical contact (Movie S1–S3). The electricity generated by the HEC‐TENGs can be stored in a capacitor for DC applications. The equivalent circuit is shown in Figure 6e; the switches control the charging/discharging operation. Figure 6f presents the contact‐mode charging curves for capacitors of different values: a 1.0 µF capacitor reaches 5 V within 70 s, whereas a 2.2 µF capacitor requires approximately 180 s to attain the same voltage. Figure 6g shows the noncontact charging capability of HEC‐TENG with a 1.0 µF capacitor. The device was set at various distances from 0 to 30 mm to examine the noncontact charging curves. As the separation distance increased, the charging rate decreased. Voltage storage of 7, 4, and 2 V was achieved within 300 s at separation distances of 1, 3, and 5 mm, respectively. In addition, noncontact energy harvesting can be used to power multiple LEDs (Movie S4). Figure 6h,i, and Movie S5 show an electronic watch powered by noncontact mode HEC‐TENG at a separation distance of 30 mm. Figure 6k,l, and Movie S6 show calculators powered under the same condition. The real‐time charging and discharging curves of the capacitor are shown in Figure 6j,m.

**FIGURE 6:**
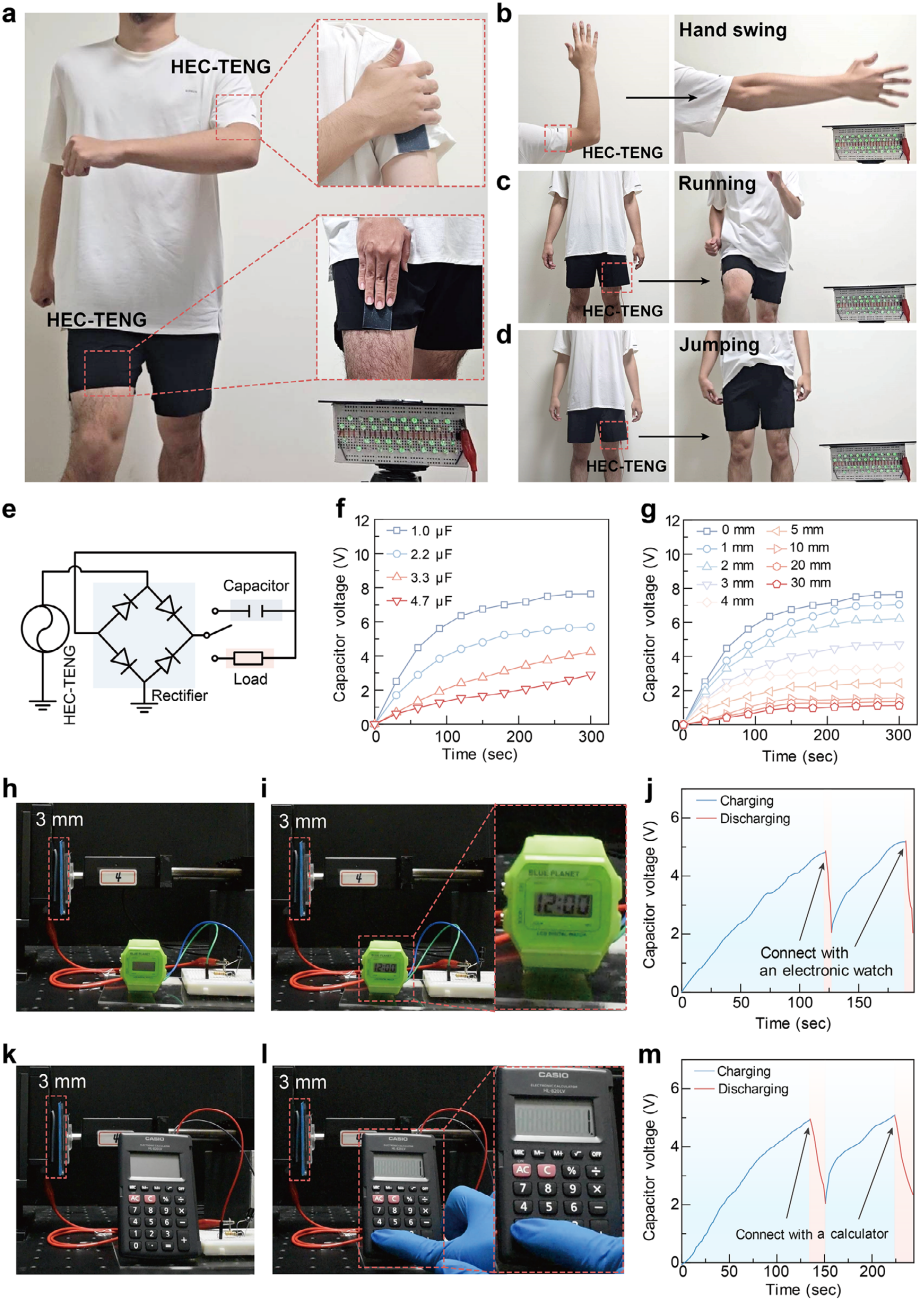
Application demonstrations of HEC‐TENG in noncontact modes. (a) Photographs of HEC‐TENG attached to the reverse side of the garment. Photographs of LEDs lighting during (b) hand swinging, (c) running, and (d) jumping. (e) Equivalent circuit of noncontact mechanical energy harvesting for charging and discharging. (f) Charging curve of capacitors with different capacitors. (g) Charging curves of capacitors at different separation distances. (h–i) Photographs of harvesting noncontact mechanical energy to power an electronic watch and (j) Corresponding charging/discharging curve. (k–l) Photographs of harvesting noncontact mechanical energy to power a calculator and (m) Corresponding charging/discharging curve.

In addition to wearable, noncontact energy harvesting, HEC‐TENGs have also been used for wearable droplet‐based and large‐area raindrop energy harvesting. As shown in Figure 7a and Movie S7, four HEC‐TENG units were integrated into the sleeve of a raincoat to harvest energy from raindrops and illuminate multiple LEDs. Thus, HEC‐TENGs can be used as IoT power sources. As shown in Figure 7b and Movie S8, the HEC‐TENGs were integrated into a droplet energy‐harvesting array to drive a multiple‐LED display screen. The charging curves for different capacitors are presented in Figure 7c, confirming the feasibility of the device as a sustainable energy source. The charging curves were systematically investigated by connecting multiple HEC‐TENG units (Figure 7d). Specifically, when 20, 30, and 40 units were connected, V_oc_ of 2.0, 3.5, and 4.8 V were achieved within 60 s, respectively. The real‐time charging curve of the HEC‐TENG was further demonstrated by modulating the water flow (turning it on and off), thereby demonstrating the charging capability of the device (Figure 7e). Furthermore, the droplet‐energy‐harvesting capability of the HEC‐TENG was demonstrated by powering small commercial electronic devices. Figure 7f,g and Movie S9 show an electronic watch powered by HEC‐TENGs integrated into the raincoat. Figure 7i,j and Movie S10 further illustrate a thermohygrometer powered by a 7 × 7 HEC‐TENG array. The corresponding real‐time charging and discharging curves of the capacitor are shown in Figure 7h,k, respectively. These demonstrations highlight the potential of the HEC‐TENG as a wearable power source and a distributed IoT energy array for raindrop energy harvesting.

**FIGURE 7:**
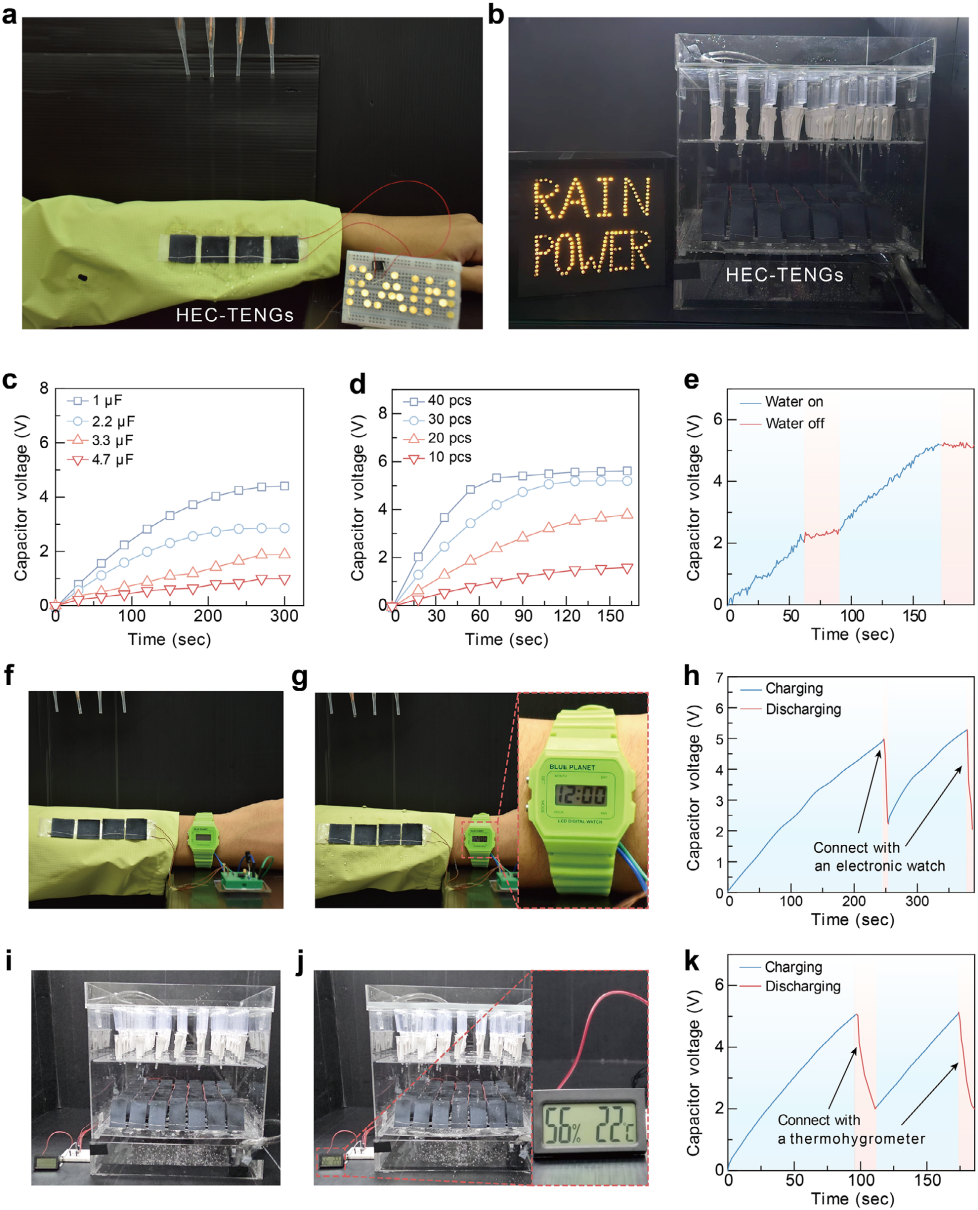
Application demonstrations of HEC‐TENGs in raindrop energy harvesting. (a) Photograph of an HEC‐TENG integrated into a raincoat for illuminating LEDs. (b) Photograph of an HEC‐TENG array illuminating LEDs. (c) Charging curves of different capacitors using HEC‐TENGs. (d) Charging curves of HEC‐TENG under different numbers of activated droplet panels. (e) Charging curves of HEC‐TENG with water flow turned on and off. (f–g) Demonstration of harvesting droplet energy to power an electronic watch, and (h) its corresponding charging/discharging curve. (i–j) Demonstration of harvesting droplet energy with an HEC‐TENG array to power a thermohygrometer, and (k) its corresponding charging/discharging curve.

## 3 Conclusion

This study proposed a large‐area, deformable noncontact TENG using HEC‐doped SR as the charge trapping and transport layer and GT as the charge storage interlayer. Benefiting from the integrated trilayer composite architecture, the HEC‐TENG exhibited excellent stretchability of up to 230% as well as scalable device dimensions (5 × 5 cm^2^). Under the contact‐separation mode, the device generated a V_oc_ of 118 V, J_sc_ of 732 µA m^−2^, and power density of 35.7 mW m^−2^, maintaining a consistent output over 5000 continuous operation cycles. Even under the noncontact mode (1 mm distance), the device generated a V_oc_ of 90 V and J_sc_ of 450 µA m^−^^2^, with a charge‐retention time of up to 470 min. Notably, the strategy could be further extended to the liquid‐solid contact mode, in which the device achieved a V_oc_ of up to 466 V and a maximum power output of 150 µW in droplet energy harvesting, highlighting its capability as a multisource energy conversion platform. Owing to its combined advantages of noncontact operation, multisource energy harvesting, and textile‐level stretchability, this device can be integrated into garments or deployed as large‐area arrays to collect energy from daily human motions and environmental stimuli, such as raindrops, for powering commercial electronics. Overall, this study demonstrates the potential of HEC materials as charge‐regulation components in triboelectric layers, paving the way for future applications of HEC‐based TENGs in wearable energy harvesting and distributed power systems.

## 4 Experimental Section

### 4.1 Materials

Tin(II) chloride (SnCl_2_, CAS No. 7772‐99‐8), titanium(IV) oxide (TiO_2_, CAS No. 13463‐67‐7), zirconium(IV) chloride (ZrCl_4_, CAS No. 10026‐11‐6), hafnium(IV) chloride (HfCl_4_, CAS No. 13499‐05‐3), strontium dichloride hexahydrate (SrCl_2_·6H_2_O, CAS No. 10025‐70‐4), niobium(V) chloride (NbCl_5_, CAS No. 10026‐12‐7), and sodium hydroxide (NaOH, CAS No. 1310‐73‐2) were purchased from Sigma‐Aldrich (St. Louis, MO, USA). The cotton textiles were purchased from a local store.

### 4.2 Fabrication of GT

Cotton textile was placed in a tube furnace under a nitrogen atmosphere (gas flow: 20 sccm) and heated to 1000 °C at a rate of 3°C min^−^^1^ for 200 min to form the carbon textile.

### 4.3 Synthesis of HEC particles

SnCl_2_ (0.15 g), TiO_2_ (0.06 g), ZrCl_4_ (0.19 g), HfCl_4_ (0.26 g), SrCl_2_·6H_2_O (1.08 g), and NbCl_5_ (0.21 g) were added into a 40 mL of DI water. NaOH was separately dissolved in 40 mL of DI water. The two solutions were then mixed and stirred for 30 min. The mixture was poured into an autoclave reactor and heated to 200°C for 2 h. After cooling, the product was collected by centrifugation and alternately rinsed three times with DI water and ethanol. The desired HEC particles were obtained after drying at 60°C.

### 4.4 Preparation of HEC‐TENG

A square groove mold with sandpaper at the bottom (5 × 5 cm^2^, 3 mm thickness) was fabricated using acrylic boards and sandpaper. Parts A and B of the SR were mixed (1:1, v/v) with HEC particles and poured into the mold as the first layer. The mixture was incubated for 30 min at room temperature. The GT was placed on the HEC‐SR as the second layer. After 30 min, parts A and B of the SR were mixed with CB and poured into the mold as a third layer. Following 30 min of incubation, the device was separated and reversed to obtain a tri‐layered HEC‐TENG.

### 4.5 Material Characterization and Electrical Measurement

Surface morphology and elemental analyses were performed using a Zeiss EVO 60 scanning electron microscope and an OXFORD X‐Max 50 mm^2^ energy‐dispersive X‐ray spectroscope. A Rigaku Ultima IV diffractometer was used to explore the crystal structure of HEC particles. TEM images of HEC particles and GT were obtained using a Tecnai G2 field‐emission transmission electron microscope. A component analysis of the materials was performed using Raman scattering spectroscopy (Tokyo Instruments Nanofinder 30). Contact and noncontact TENG tests were performed using a linear motor (LinMot, E1100). The applied force was measured using a dual‐range force sensor (Vernier LabQuest Mini). The V_out_ output curves were measured using a WaveSurfer 510 digital oscilloscope. V_oc_, Q_tr_, and charging curves were measured using a Keithley 6514 electrometer. The I_sc_ and J_sc_ were measured using a Stanford low‐noise current amplifier (SR570). The surface potential was measured using a Trek Model 541A Electrostatic Voltmeter.

## Conflicts of Interest

The authors declare no conflicts of interest.

## Supporting information




**Supporting File 1**: adma74157‐sup‐0001‐SuppMat.docx.

**Supporting File 2**: adma74157‐sup‐0002‐MovieS1.mp4.

**Supporting File 3**: adma74157‐sup‐0003‐MovieS2.mp4.

**Supporting File 4**: adma74157‐sup‐0004‐MovieS3.mp4.

**Supporting File 5**: adma74157‐sup‐0005‐MovieS4.mp4.

**Supporting File 6**: adma74157‐sup‐0006‐MovieS5.mp4.

**Supporting File 7**: adma74157‐sup‐0007‐MovieS6.mp4.

**Supporting File 8**: adma74157‐sup‐0008‐MovieS7.mp4.

**Supporting File 9**: adma74157‐sup‐0009‐MovieS8.mp4.

**Supporting File 10**: adma74157‐sup‐0010‐MovieS9.mp4.

**Supporting File 11**: adma74157‐sup‐00011‐MovieS10.mp4.

## Data Availability

The data that support the findings of this study are available from the corresponding author upon reasonable request.
