# Supplementary material for: High‐Entropy Ceramic‐Enhanced Deformable Triboelectric Nanogenerator for Noncontact Biomechanical and Raindrop Energy Harvesting
Source: Adv Mater. 2026 Jul 25;38(58):e74157. doi: 10.1002/adma.74157 (PMC13674617; doi:10.1002/adma.74157)
Supplement: Supplementary file 1 [file ADMA-38-e74157-s003.docx]

Supporting information

**High-entropy ceramic-enhanced deformable triboelectric nanogenerator for noncontact biomechanical and raindrop energy harvesting**

Shengyou Li^1,2,†^, Wei-Chen Peng^1, †^, Syun-Hong Chou^3^, Zhi-Xian Yan^1^, Jiann-Yeu Chen^4^, Dun-Jie Jhan^5^, Wei-Chun Yang^1^, Shi-Hong Chen^1^, Yi-Lin Huang^1^, Cheng-Hung Tsai^1^, Ming-Yen Lu^5^, Heng-Jui Liu^1,^*, Cheolmin Park^2,^*, and Ying-Chih Lai^1,4,6,^*

*^1^Department of Materials Science and Engineering, National Chung Hsing University, Taichung, 402202, Taiwan*

*^2^Department of Materials Science and Engineering, Yonsei University, Seoul 03722, Republic of Korea*

*^3^Department of Materials Science and Engineering, National Yang Ming Chiao Tung University, Hsinchu, 30010, Taiwan*

*^4^Innovation and Development Center of Sustainable Agriculture, i-Center for Advanced Science and Technology, National Chung Hsing University, Taichung, 402202, Taiwan*

*^5^Department of Materials Science and Engineering, National Tsing Hua University, Hsinchu, 300, Taiwan*

*^6^Department of Physics, National Chung Hsing University, Taichung City, 402202, Taiwan*

*Corresponding authors: Heng-Jui Liu ([hengjui0109@dragon.nchu.edu.tw](mailto:hengjui0109@dragon.nchu.edu.tw)); Cheolmin Park (*[cmpark@yonsei.ac.kr](mailto:cmpark@yonsei.ac.kr)*); Ying-Chih Lai ([yclai@nchu.edu.tw](mailto:yclai@nchu.edu.tw))*

*^†^Shengyou Li and Wei-Chen Peng contributed equally to this work.*

Table S1. Summary of recent TENGs for noncontact or droplet energy harvesting

| **TENG materials** | **Electrode** | **Stretchability** | **Performance output** | | | **Ref.** |
| --- | --- | --- | --- | --- | --- | --- |
|  |  |  | **Contact-separation mode** | **Noncontact mode** | **Liquid-solid contact mode** |  |
| HEC-doped sillon rubber/graphene-like textile/carbon black | Carbon black/silicone rubber | 230% strain (entire device) | V_oc_ = 140 V;  J_sc_ = 1.87 mA m^-2^;  P_d_ = 32.5 mW m^-2^  at 4 Hz | V_oc_ = 90 V, J_sc_ = 450 µA m^-2^ at 1mm distance; 470 min charge retention | V_oc_ = 466 V;  J_sc_ = 254.8 µA m^-2^  ;  P_d_= 60 mW m^-2^ with tap water droplet | This work |
| Graphite-like composite/  graphite-like textile/silicone rubber | Graphite-like textile/silicone rubber | 200% strain | V_oc_ = 288 V;  J_sc_ = 0.492 mA m^-2^;  P_d_ = 34.4 mW m^-2^  at 4 Hz | V_oc_ = 186.5 V at 1mm distance | × | [1] |
| Topological insulator/silicone rubber/graphite-like carbonized textile | AgNWs-based fabric | 100% strain | V_oc_ = 452 V;  J_sc_ = 1.96 mA m^-2^;  P_d_ = 179 mW m^-2^  at 4 Hz | V_oc_ = 94 V,  J_sc_ = 23 μA m^−2^  at 1 cm, 3 Hz  10000 min charge retention | × | [2] |
| Siloxene/Ecoflex /MoS_2_/laser-induced graphene | Copper | × | V_oc_ = 38.4 V,  J_sc_ = 9.5 μA m^-2^,  P_d_= 60 mW m^-2^  at 3 Hz | V_oc_ = 38 V,  J_sc_ = 12 μA m^−2^  at 1 mm distance  6000 min charge retention | × | [3] |
| Co-nanoporous carbon /Ecoflex | Mxene/  Ecoflex | × | V_oc_ = 33 V,  J_sc_ = 16.1 μA m^-2^,  P_d_= 3 mW m^-2^  at 3 Hz | V_oc_ = 32 V,  J_sc_ = 13.2 μA m^−2^  at 2 cm distance  4,320 min charge retention | × | [4] |
| PI-b-poly(styrene-co-NC60methylstyrene) | AgNWs  /PDMS/Al | × | V_oc_ = 505.1 V,  J_sc_ = 117.9 mA m^-2^,  P_d_= 1.95 mW m^-2^  at 3 Hz | V_oc_ = 2.5 V at 1mm distance  V_oc_ =0.53 V at 4 mm distance | × | [5] |
| Graphene oxide/ Ecoflex | Al | × | × | V_oc_ = 0.8 V at 5.5mm distance  V_oc_ = 0.3 V at 12.7 mm distance | × | [6] |
| Silicone rubber/AgNWs/PDMS | Liquid metal | 58.4% strain | V_oc_ = 11.35 V | V_oc_ = 7 V at 2.5 mm | × | [7] |
| Mxene(Ti_3_C_2_T_x_)/Silicone nanocomposite layer | Conductive sponge/PET film | × | V_oc_ = 251.8 V,  J_sc_ = 687.5 μA m^-2^, 2 Hz | V_oc_ = 17.5 V,  J_sc_ = 17.8 μA m^-2^  at 20 mm, 2 Hz | × | [8] |
| Fluorinated ethylene propylene (FEP)/Fish bladder film | Cu | × | V_oc_ = 106 V,  J_sc_ = 4562.5 μA m^-2^,  P_d_ = 200 mW m^-2^ at 3Hz | V_oc_ =72.8 V at 1 mm, 3 Hz | × | [9] |
| Poly(tetrafluoroethylene) (PTFE) | Cu | × | V_oc_ = 2 V,  J_sc_ = 12.5 nA m^-2^, 1 Hz | V_oc_ =1.81 V  J_sc_ = 10.3 μA m^-2^  at 10 cm | × | [10] |
| Gelatin/nanoporous carbon gel | Gelatin/PEDOT:PSS gel | 400% strain | × | V_oc_ = 0.64 V,  J_sc_ = 12.5 nA m^-2^ at 1cm | × | [11] |
| 3D printed MXene/CNF aerogel | Cu | × | × | V_oc_ = 25 V at 1cm,  V_oc_ = 3.5 V at 50cm, | × | [12] |
| PAN/PVDF/ BaTiO_3_/PEDOT:PSS film | Cu | × | V_oc_ = 300 V,  J_sc_ = 1.93 μA m^-2^  P_d_ = 225 μW cm^-2^  at 3 Hz | V_oc_ = 20 V, J_sc_ = 125 μA m^-2^ at 5 cm, 3 HZ | × | [13] |
| FEP/[(FeNiCrMn)_0.8_Co_0.2_]_3_O_4_-doped PDMS | Cu | × | × | × | V_oc_ = 420 V;  J_sc_ = 14.6 μA cm^-2^ | [14] |
| FEP/(MgCoNiZn)_1-x_Cu_x_O-doped PDMS | Cu | × | × | × | V_oc_ = 525 V | [15] |
| PTFE/ITO | Al | × | × | × | V_oc_ = 143.5 V  P_d =_ 50.1 W m^-2^ | [16] |
| Biommetric structured PDMS-Graphene film | Cu | × | × | × | V_oc_ = 20 V  I_sc =_ 400 nA | [17] |
| E-PTFE film | Al | × | × | × | V_oc_ = 300 V  P_d =_ 8 W m^-2^ | [18] |
| FEP | Conductive water electrode | × | × | × | V_oc_ = 80 V  P_d =_ 464 mW m^-2^ | [19] |
| PDMS | Conductive hydrogel | 70% strain | × | × | V_oc_ = 45 V  P_d =_ 11.7 mW m^-2^ | [20] |

**Figure S1.** Stress-strain curve of the deformable HEC-TENG.

**Figure S2.** SEM image of the HEC/SR triboelectric layer peeled off from the sandpaper substrate, showing a rough surface morphology.

**Figure S3.** Side-view SEM image showing HEC particles are uniformly distributed within the SR matrix

**Figure S4.** High-resolution XPS spectra of HEC NPs. **a**: Sr 3d, **b**: Ti: 2p, **c** Zr: 3d, **d**: Hf 4f, **e**: Sn 3d, and **f**: Nb 3d.

**Figure S5.** SEM images and corresponding EDS analysis of a) cotton textile and b) graphite-like textile.

**Figure S6.** Comparison of the dielectric constants of pristine SR, Al₂O₃-doped SR, BaTiO₃-doped SR, and TiO₂-doped SR composites. The filler content was fixed at 0.3 wt% for all ceramic-doped SR samples. The dielectric constant was measured at a frequency of 1 kHz.

**Figure S7.** (a) Cross-sectional SEM images of the HEC-doped SR layer with different HEC filler contents of 0.1, 0.3, 0.5, and 0.7 wt%. (b) Stress-strain curves of HEC-doped SR composites with different HEC filler contents of 0, 0.1, 0.2, 0.3, 0.4, 0.5, 0.7 and 1.0 wt%.

**Figure S8.** a) V_out_ and b) J_sc_ output curves of HEC-doped SR/GT TENGs with different HEC weight ratio obtained by Occilloscope.

**Figure S9.** a) V_oc_ and b) Q_tr_ output curves of HEC-doped SR/GT TENGs with different HEC weight ratio obtained by Keithley 6514.

**Figure S10.** Comparison of the time-dependent output curves of pristine SR, Al₂O₃-doped SR, BaTiO₃-doped SR, and TiO₂-doped SR composites with a fixed ceramic filler content of 0.3 wt%, including (a) V_out_ obtained by oscilloscope; (b) J_sc_ obtained by SR 570; (c) V_oc_ and (d) Q_tr_ obtained by Keithley 6415.

**Figure S11.** a) V_out_ and b) J_sc_ output with different device configurations: SR, HEC-doped SR, SR/GT, and HEC-doped SR/GT.

**Figure S12.** a) V_oc_ and b) Q_tr_ output with different device configurations: SR, HEC-doped SR, SR/GT, and HEC-doped SR/GT.

**Figure S13.** a) V_oc_, c) J_sc_, and e) Q_tr_ output of HEC-TENG with different RHs (30% to 90%) and b, d, f) corresponding linear fitting curves.

**Figure S14.** a) V_oc_, c) J_sc_, and e) Q_tr_ output of HEC-TENG with different temperatures (303 to 343 K) and b, d, f) corresponding linear fitting curves.

**Figure S15.** a) V_oc_ and b) Q_tr_ performance output under different contact frequencies (1–4 Hz).

**Figure S16.** Linear sensitivity fitting of V_out,p-p_ and J_sc,peak_ values under different contact forces (0–10 N).

**Figure S17.** (a) Response time of the HEC-TENG used as a self-powered pressure sensor. (b) Continuous V_oc_ curves of the HEC-TENG during 6000 repeated pressure cycles. Applied pressure is 4 kPa.

**Figure S18.** V_oc_ and Q_tr_ output in continuous operation from 1 to over 5000 cycles.

**Figure S19.** Comparison of the V_out,p-p_ values generated by the HEC-TENG during contact-separation with different materials.

**Figure S20.** Schematic illustration of the pre-charging process and the noncontact operating mechanism of the HEC-TENG.

Prior to noncontact TENG measurements, the device is first operated in a contact-separation mode for several cycles (~10 cycles), allowing the HEC-doped SR layer to capture triboelectric charges, which subsequently accumulate in the GT layer. After the output voltage and current can generate a stable output, the operation is switched to the noncontact mode. When the nitrile layer is approaching the HEC-doped SR layer, electrons flow from the external circuit to the SR/CB layer; when the nitrile layer is separating, electrons flow back to the external circuit, thereby enabling cyclic noncontact triboelectric output. In terms of the voltage output, the V_oc_ increased as the nitrile layer separated from the HEC-doped SR layer and reached its maximum value at the maximum separation distance. As the nitrile layer approached the HEC-doped SR layer again, the V_oc_ gradually decreased and returned to zero when the preset gap distance was reached. Upon repeated cycling of this process, the V_oc_ output maintained a stable output.

**Figure S21.** a) V_oc_, b) J_sc_, and c) Q_tr_ output of noncontact HEC-TENG with different separation distance from 0-3 cm.

**Figure S22.** Long-term cyclic V_oc_ output of the HEC-TENG in non-contact mode measured at a fixed separation distance of 0.3 cm and a frequency of 3 Hz.

**Figure S23.** Photographs of the experimental setup for droplet energy harvesting using the HEC-TENG, including images (a) before droplet release, (b) after droplet impact under bright conditions, and (c) under dark conditions.

**Figure S24.** a) Q_tr_ and b) I_sc_ output of HEC-DEG among different droplet volumes (10–80 µL).

**Figure S25.** I_sc_ output of HEC-DEG with different water droplet a) height and b) tilt angles.

**Figure S26.** (a) Photograph of the HEC-TENG attached to the inner side of the trousers at the knee region for harvesting biomechanical energy from knee bending during running. (b) Corresponding V_oc_ output curve.

**References**

1. X. Peng, W.-C. Peng, Y.-T. Chen, et al., “Synergistically Designed Carbon-Based Hybrid Non-Contact Triboelectric-and-Electromagnetic Nanogenerator with Ultralong Charge Retention for Wearable and Ambient Electromagnetic-Waste Energy Harvesting and Self-Powered Sensing,” *Materials Science and Engineering R-Reports* 165 (2025): 100994, <https://doi.org/10.1016/j.mser.2025.100994>.

2. S.-H. Chou, Y.-T. Chen, Z.-X. Yan, et al., “Topological-Insulator Nanocomposite and Graphite-Like Tribo-Charge-Accumulating Fabric Enabling High-Performance Non-Contact Stretchable and Textile-Based Triboelectric Nanogenerators with Robust Charge Retention,” *Advanced Energy Materials* 14 (2024): 2402169, <https://doi.org/10.1002/aenm.202402169>.

3. K. Shrestha, S. Sharma, G. B. Pradhan, et al., “A Siloxene/Ecoflex Nanocomposite-Based Triboelectric Nanogenerator with Enhanced Charge Retention by Mos2/Lig for Self-Powered Touchless Sensor Applications,” *Advanced Functional Materials* 32 (2022): 2113005, <https://doi.org/10.1002/adfm.202113005>.

4. S. M. S. Rana, M. A. Zahed, M. T. Rahman, et al., “Cobalt-Nanoporous Carbon Functionalized Nanocomposite-Based Triboelectric Nanogenerator for Contactless and Sustainable Self-Powered Sensor Systems,” *Advanced Functional Materials* 31 (2021): 2105110, <https://doi.org/10.1002/adfm.202105110>.

5. J. W. Lee, S. Jung, J. Jo, et al., “Sustainable Highly Charged C60-Functionalized Polyimide in a Non-Contact Mode Triboelectric Nanogenerator,” *Energy & Environmental Science* 14 (2021): 1004-1015, <https://doi.org/10.1039/D0EE03057K>.

6. Y. Tang, H. Zhou, X. Sun, et al., “Triboelectric Touch-Free Screen Sensor for Noncontact Gesture Recognizing,” *Advanced Functional Materials* 30 (2020): 1907893, <https://doi.org/10.1002/adfm.201907893>.

7. W. Liu, Y. Duo, J. Liu, et al., “Touchless interactive Teaching of Soft Robots through Flexible Bimodal Sensory Interfaces,” *Nature Communications* 13 (2022): 5030, <https://doi.org/10.1038/s41467-022-32702-5>.

8. C. Zhao, Z. Wang, Y. Wang, et al., “Mxene-Composite-Enabled Ultra-Long-Distance Detection and Highly Sensitive Self-Powered Noncontact Triboelectric Sensors and Their Applications in Intelligent Vehicle Perception,” *Advanced Functional Materials* 33 (2023): 2306381, <https://doi.org/10.1002/adfm.202306381>.

9. J. Ma, J. Zhu, P. Ma, et al., “Fish Bladder Film-Based Triboelectric Nanogenerator for Noncontact Position Monitoring,” *ACS Energy Letters* 5 (2020): 3005-3011, <https://doi.org/10.1021/acsenergylett.0c01062>.

10. S. Peng, Y. Feng, Y. Liu, et al., “New Blind Navigation Sensor Based on Triboelectrification and Electrostatic Induction,” *Nano Energy* 104 (2022): 107899, <https://doi.org/10.1016/j.nanoen.2022.107899>.

11. P. Wang, Y. Lv, J. Duan, et al., “A Thermally Responsive Phase-Change Hydrogel for Skin-Mountable Multifunctional Sensors,” *Nano Energy* 136 (2025): 110722, <https://doi.org/10.1016/j.nanoen.2025.110722>.

12. B. Wang, M. Gao, X. Fu, et al., “3d Printing Deep-Trap Hierarchical Architecture-Based Non-Contact Sensor for Multi-Direction Motion Monitoring,” *Nano Energy* 107 (2023): 108135, <https://doi.org/10.1016/j.nanoen.2022.108135>.

13. R. Cao, L. Yang, W. Hu, et al., “Janus-Structured Teng for Complex Dual-Mode Communication Using Water Medium across 200 M,” *Advanced Functional Materials* 36 (2026): e08124, <https://doi.org/10.1002/adfm.202508124>.

14. Y. Zhou, Y. Zeng, J. Wang, et al., “Enhancement of the Voltage Output of Droplet Electricity Generators Using High Dielectric High-Entropy Oxide Composites,” *Energy & Environmental Science* 17 (2024): 3580-3593, <https://doi.org/10.1039/D4EE01234H>.

15. C. Wang, J. Wang, P. Wang, et al., “High-Entropy Ceramics Enhanced Droplet Electricity Generator for Energy Harvesting and Bacterial Detection,” *Advanced Materials* 36 (2024): 2400505, <https://doi.org/10.1002/adma.202400505>.

16. W. Xu, H. Zheng, Y. Liu, et al., “A Droplet-Based Electricity Generator with High Instantaneous Power Density,” *Nature* 578 (2020): 392-396, <https://doi.org/10.1038/s41586-020-1985-6>.

17. W. Zhou, X. Zhu, Z. Quan, et al., “Bioinspired Dual-Function Device Integrating Fog Harvesting and Hydro-to-Electricity Conversion for Sustainable Supply of Freshwater and Electricity,” *Advanced Functional Materials* 35 (2025): 2426068, <https://doi.org/10.1002/adfm.202426068>.

18. F. Li, J. Zhao, B. Li, et al., “Water-Triboelectrification-Complemented Moisture Electric Generator,” *ACS Nano* 18 (2024): 30658-30667, <https://doi.org/10.1021/acsnano.4c09581>.

19. S. Jang, S. A. Shah, J. Lee, et al., “Beyond Metallic Electrode: Spontaneous Formation of Fluidic Electrodes from Operational Liquid in Highly Functional Droplet-Based Electricity Generator,” *Advanced Materials* 36 (2024): 2403090, <https://doi.org/10.1002/adma.202403090>.

20. S. Jang, S. Lee, S. A. Shah, et al., “Hydrogel-Based Droplet Electricity Generators: Intrinsically Stretchable and Transparent for Seamless Integration in Diverse Environments,” *Advanced Functional Materials* 35 (2025): 2411350, <https://doi.org/10.1002/adfm.202411350>.
